# How Autoantibodies Regulate Osteoclast Induced Bone Loss in Rheumatoid Arthritis

**DOI:** 10.3389/fimmu.2019.01483

**Published:** 2019-07-03

**Authors:** Ulrike Steffen, Georg Schett, Aline Bozec

**Affiliations:** Department of Internal Medicine 3, University of Erlangen-Nuremberg, Erlangen, Germany

**Keywords:** rheumatoid factor (RF), autoantibodies against citrullinated proteins (ACPA), osteoclasts, rheumatoid arthritis, cytokines

## Abstract

Rheumatoid arthritis (RA) is a chronic inflammatory disease, characterized by autoimmunity that triggers joint inflammation and tissue destruction. Traditional concepts of RA pathogenesis have strongly been focused on inflammation. However, more recent evidence suggests that autoimmunity *per se* modulates the disease and in particular bone destruction during the course of RA. RA-associated bone loss is caused by increased osteoclast differentiation and activity leading to rapid bone resorption. Autoimmunity in RA is based on autoantibodies such as rheumatoid factor (RF) and autoantibodies against citrullinated proteins (ACPA). These autoantibodies exert effector functions on immune cells and on bone resorbing osteoclasts, thereby facilitating bone loss. This review summarizes potential pathways involved in increased destruction of bone tissue in RA, particularly focusing on the direct and indirect actions of autoantibodies on osteoclast generation and function.

## Introduction

Skeletal homeostasis is maintained by continuous removal and replacement of bone throughout life. This process is controlled by the coordinated activity of specific bone cells. Osteoclasts are highly specialized multinucleated cells derived from hematopoietic precursors of the myeloid lineage with the capacity to resorb bone [reviewed in Tanaka et al. (1)]. Osteoclast formation is controlled by the action of soluble mediators, such as receptor activator of nuclear factor-κB ligand (RANKL; also known as TNFSF11), macrophage colony-stimulating factor 1 (M-CSF), and negative regulators, such as the decoy receptor for RANKL, osteoprotegerin (OPG). These cytokines are provided by cells of the osteoblast lineage and immune cells located within the bone microenvironment [reviewed in Schett (2)]. Bone resorption also liberates growth factors deposited in bone, which can act locally on osteoblasts and immune cells.

In parallel to osteoclast-mediated bone resorption, bone formation results from the proliferation of skeletal stem cells and their differentiation into osteoblast. Their fate is to either stay as bone lining cells or to be embedded into the bone matrix as osteocytes [reviewed in Bonewald (3)]. The osteoblast cell lineage includes osteoblast precursors, bone lining cells and osteocytes. Each of them express specific signals that regulate resident cells within the bone marrow. In addition to the crosstalk between different types of bone cells, there is a tight interaction between bone and immune cells, which is still not fully characterized. The importance of this interaction is reflected by diseases, such as rheumatoid arthritis (RA), in which immune activation is linked to bone loss.

RA is a chronic systemic autoimmune disease affecting about 1% of the population worldwide [reviewed in McInnes and Schett (4)]. It is associated with pain, joint swelling, progressive disability and systemic comorbidity. One of the major consequences of RA is the degradation of cartilage and bone tissue. This process results in joint destruction, which leads to significant loss of life quality for the patients. RA-associated bone loss is characterized by three different manifestations: (i) local erosions in the inflamed joints, where bone and cartilage are in direct contact with the inflamed synovium, (ii) periarticular bone loss of trabecular and cortical bone close to sites of inflammation, and (iii) systemic osteopenia and osteoporosis [reviewed in Zerbini et al. (5)]. All three forms of bone loss are caused by altered bone homeostasis with increased osteoclast generation and activity resulting in accelerated bone resorption, while osteoblast-mediated bone formation is suppressed. The reasons for enhanced osteoclast activity have been in the focus of extensive research. Aside from direct inter-cellular interactions and systemic effects of inflammatory cytokines, autoantibodies have been found to play a major role both via directly influencing osteoclasts, as well as, through the induction of inflammatory cytokines released by macrophages.

In this review, we will summarize the current knowledge on autoantibody-mediated bone loss in RA. We will focus on the direct effects of autoantibodies on osteoclasts and pre-osteoclasts as well as indirect effects via cytokines released by activated macrophages. In addition, we will discuss the implications of antibody glycosylation.

## The Regulation of Osteoclast Activity and Differentiation by Autoantibodies

### Autoantibodies in RA

Although the causes of RA are diverse and not completely understood, it is clear that disease specific autoantibodies constitute an important trigger. The main autoantibodies associated with RA are the rheumatoid factor (RF) and autoantibodies against citrullinated proteins (ACPA). RF is directed against the Fc part of IgG and mainly occurs as IgM. However, to a smaller extent, RF can also be detected as IgG or IgA. Up to 70% of RA patients are RF positive. Of note, RF is also found in a subset of healthy people, especially in the elderly, in patients with other rheumatic diseases (e.g., Sjögren's syndrome or systemic lupus erythematosus) or in patients with viral infections like hepatitis C (6, 7). Although RF is positively associated with increased bone erosion, especially in ACPA positive patients (8, 9), there are no data available about its direct effects on cytokine production or osteoclastogenesis. As RF is directed against IgG, it might lead to a constant basal inflammation by the formation of random IgG complexes or enhance the size of existing immune complexes formed by other autoantibodies. Indeed, the addition of monoclonal IgM-RF increased the production of the pro-inflammatory cytokine TNF-α by macrophages after treatment with ACPA-containing IgG from RA patients (10), suggesting a synergistic interaction of ACPA and RF.

In contrast to RF, ACPA are highly specific for RA with a very low prevalence in healthy people (11, 12). ACPA provide diagnostic value in predicting disease severity and the likelihood to develop bone erosions in RA patients (13). ACPA are detectable up to 10 years before clinical onset of RA (14). Some months before the occurrence of clinical symptoms, ACPA broaden their epitope recognition and isotype usage profile and change their glycosylation toward a more inflammatory phenotype (14–16). In 2010, ACPA have been included into the diagnostic criteria for RA by the American College of Rheumatology (ACR) and European League Against Rheumatism (EULAR) (17). ACPA recognize a variety of citrullinated proteins with citrullinated vimentin, α-enolase, fibrinogen and collagen being the most prominent antigens. Citrullination is a post-translational modification of a positively charged arginine residue into a partially negatively charged citrulline residue. As this process changes the net charge of a protein, neo epitopes appear, that can be recognized by the immune system resulting in autoantibody formation. Citrullination is performed by enzymes of the peptidylarginine deiminase (PAD) family and occuring physiologically during the formation of neutrophil extracellular traps (NETs), apoptosis and skin keratinization [reviewed in Baka et al. (18)]. In addition, the bacterium *Porphyromonas gingivalis* (which is involved in periodontitis) releases PAD (19). Bacterial PAD is suspected to contribute to protein citrullination and ACPA formation, but more research is needed to truly confirm a relationship between periodontitis and RA [reviewed in Araujo et al. (20) and Potempa et al. (21)]. Another trigger of citrullination, especially in the lung, is smoking [reviewed in Klareskog et al. (22)].

Apart from ACPA, a couple of other autoantibodies against posttranslational modifications (AMPA) have been found in the last years, such as autoantibodies against carbamylated proteins (anti-CarP) (23) or autoantibodies against acetylated proteins (24). All groups of autoantibodies can be detected independently of each other in patients with RA. According to a meta-analysis evaluating 25 studies, ACPA are present in 47–88% of RA patients (13). Anti-CarP could be detected in 39–58% of RA patients and in 8–16% of RA patients that are ACPA negative (23, 25, 26), but also in about 7% of osteoarthritis patients and 3,6% of healthy controls (11).

### Epidemiological Evidence for Autoantibody-Mediated Bone Loss in RA

Bone loss is strongly associated with ACPA positivity in RA patients (27–29). Higher ACPA titers correlate with increased systemic osteopenia, indicating that ACPA might contribute to bone loss, either directly or via increased systemic inflammation. In the last years, several studies tried to disentangle direct ACPA-mediated effects from inflammation with inconclusive results. Llorente et al. described that the presence of ACPA was associated with baseline bone mass independently of disease activity in a cohort of early RA patients (30), suggesting direct effects of ACPA on the bone. This was further confirmed by studies describing that ACPA positive individuals without clinical signs of RA display signs of bone loss in metacarpal joints (31, 32). However, subclinical inflammation can't be fully excluded in these studies. Ten Brinck et al. reported that ACPA positive RA patients only exhibited bone resorption in the presence of local inflammation (33). However, general inflammation alone seems insufficient to induce bone loss, since patients with ACPA positive RA displayed the most severe form of bone loss when compared to patients suffering from other inflammatory diseases like seronegative RA, psoriatic arthritis or inflammatory bowel disease (34). These studies indicate that an interplay of direct and indirect effects of ACPA on bone homeostasis leads to local and systemic bone loss. We will discuss the mechanisms by which ACPA affect bone later in this review.

Like ACPA, anti-CarP are associated with higher disease severity and increased bone erosion (23, 26, 35), but more research is needed to elucidate its underlying mechanisms. The fact that ACPA fine specificity does not seem to correlate with disease progression and bone erosion (36, 37) strongly suggests common mechanisms for all AMPA to mediate bone loss, most likely via the conserved Fc part of IgG.

### FcγR Signaling in Immune Cells and in Osteoclasts

**Figure 1:**
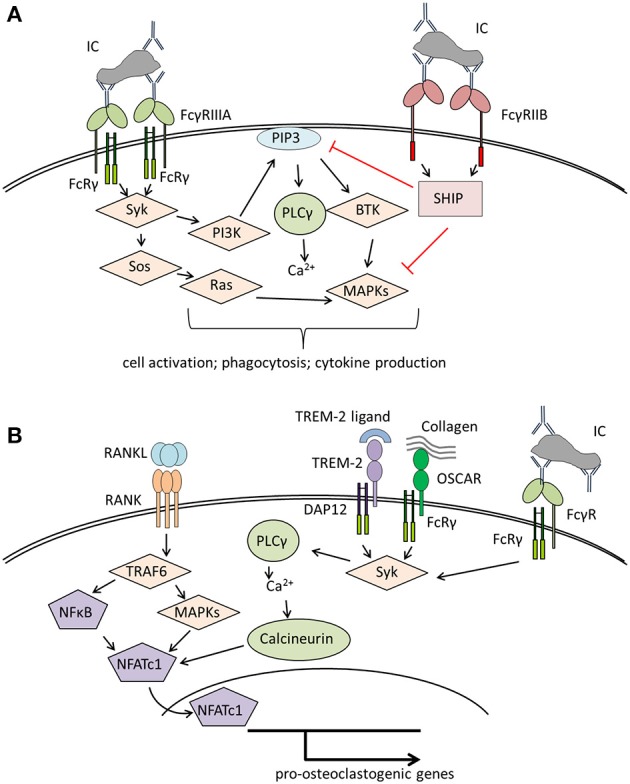
Overview of signaling pathways of **(A)** Fcγ receptors (FcγRs) on immune cells and **(B)** co-stimulatory molecules involved in osteoclastogenesis. **(A)** Crosslinking of activating FcγR (here FcγRIIIA) results in Syk activation starting various signaling pathways that lead to immune cell activation and effector functions like phagocytosis or cytokine production. The distinct signaling pathways have been reviewed in detail in Nimmerjahn and Ravetch (40), Rosales (42). **(B)** Binding of RANKL to RANK leads to the activation of TRAF6, NFκB, and several MAP kinases resulting in the activation of NFATc1, the master transcription factor for pro-osteoclastogenic genes. For a stable NFATc1 activation, costimulatory signals provided by several receptors associated to the accessory molecules DAP12 or FcRγ, like TREM-2 or OSCAR are needed [reviewed in detail in Humphrey and Nakamura (43)]. These receptors lead to Syk activation with subsequent calcium influx enhancing NFATc1 activation. In a similar way, binding of immune complexes to FcγR initiates co-stimulatory signals, thereby enhancing osteoclastogenesis. BTK, Bruton's tyrosin kinase; DAP12, DNAX activation protein of 12kDa; FcRγ, Fc receptor gamma chain; IC, immune complex; MAPKs, mitogen-activated protein kinases; NFATc1, nuclear factor of activated T cells cytoplasmic 1; NFκB, nuclear factor kappa-light-chain-enhancer of activated B cells; OSCAR, Osteoclast-associated immunoglobulin-like receptor; PI3K, phosphatidylinositol-3 kinase; PIP3, phosphatidylinositol 3,4,5-trisphosphate; PLCγ, phospholipase Cγ; SHIP, SH2 domain-containing inositol 5'-phosphatase; Syk, spleen tyrosine kinase; Sos, son of Sevenless; TRAF6, TNF receptor associated factor 6; TREM-2, triggering receptor expressed on myeloid cells 2.

Humans possess five classical FcγR: FcγRI, FcγRIIA, FcγRIIB FcγRIIIA, and FcγRIIIB that differ in their IgG binding capacity and downstream signaling pathways [reviewed in Nimmerjahn and Ravetch (38), Ghazizadeh (39), Nimmerjahn and Ravetch (40), and Ono (41)]. FcγRI is the only known high-affinity FcγR that is able to bind uncomplexed IgG while all other FcγR need the crosslinking effects of immune complexes to become activated. Activation of FcγRI, FcγRIIA and FcγRIIIA results in the phosphorylation of either an intrinsic immunoreceptor tyrosine-based activation motif (ITAM) domain (as for FcγRIIA) or an ITAM domain supplied by accessory proteins, typically the Fc-receptor common γ-chain (FcRγ-chain) (Figure 1A). This phosphorylation leads to the recruitment and activation of spleen tyrosine kinase (Syk) and its downstream targets. The most important events after FcγR activation are calcium influx and the engagement of the rat sarcoma (RAS)- rapidly accelerated fibrosarcoma (RAF)- mitogen-activated protein kinase (MAPK) pathway, resulting in antigen uptake, phagocytosis, cellular activation, and the release of pro-inflammatory cytokines by immune cells. Activating FcγRs have one potent inhibitory opponent: FcγRIIB, which contains an intrinsic immunoreceptor tyrosine-based inhibition motif (ITIM) domain. The ITIM domain interferes with ITAM signaling through engagement of src homology 2-containing inositol phosphatase (SHIP) or src homology 2 domain-containing protein tyrosine phosphatases (SHPs) that inhibit calcium influx by hydrolyzing phosphoinositide intermediates. FcγRIIIB, expressed on neutrophils, has a glycosylphosphatidylinositol (GPI) anchor without a signaling domain (44). The mechanisms by which FcγRIIIB transduces signals are still unknown.

Osteoclasts belong to the myeloid cell lineage and share many features with macrophages. Like macrophages, osteoclasts and their precursors express FcγR (45–47) with FcγRI, FcγRIIB and FcγRIIIA being significantly upregulated during human *ex vivo* osteoclastogenesis (46). It is not clear whether FcγR possess a role in bone homeostasis. However, activation of FcγR with crosslinked antibodies enhanced osteoclastogenesis from murine bone marrow cells (47). This suggests that FcγR regulate osteoclast activity and bone resorption. Of note, osteoclast development is strongly dependent on co-stimulatory signals provided by the accessory protein FcRγ-chain (that is also used by FcγR) and its functional analog DNAX activation protein of 12 kDa (DAP12) (Figure 1B). Mice lacking both proteins display a severe osteopetrotic phenotype with impaired osteoclast function (48, 49). FcRγ-chain is likely involved in osteoblast-osteoclast and osteoclast-matrix interactions as it is associated with paired immunoglobulin-like receptor A (PIR-A) and osteoclast-associated receptor (OSCAR) (48, 50). DAP12 associates with TREM-2 and signal-regulatory protein b1 (SIRPb1), which seems to be necessary for the communication between osteoclast precursors (48, 51). Both accessory proteins might enhance the effects of RANKL-signaling by amplifying calcium influx required for the activation of the pro-osteoclastogenic transcription factor, NFATc1 (48).

### Direct Actions of ACPA on Osteoclastogenesis

The described positive effects of FcγR signaling on osteoclastogenesis suggest that autoantibodies or autoimmune complexes could directly enhance osteoclast development and hence osteoclast-mediated bone loss in patients with RA. Indeed, we found that affinity-purified autoantibodies against citrullinated vimentin from RA patients, but not ACPA-depleted serum IgG were able to enhance osteoclastogenesis and bone resorption in *ex vivo* osteoclastogenesis assays as well as in recombination activation gene 1 (RAG1)-deficient mice (52). This effect was based on direct binding of autoantibodies to osteoclasts and their precursors resulting in the release of the pro-inflammatory cytokine TNF-α. In later studies, Krishnamurthy and colleagues suggested similar pro-osteoclastogenic effects of ACPA using polyclonal ACPA, purified with a cyclic citrullinated peptide (CCP)-column as well as monoclonal ACPA (53). While the results with polyclonal anti-CCP antibodies confirmed the original findings with polyclonal antibodies against citrullinated vimentin, the monoclonal antibody preparations were later demonstrated to not recognize citrullinated proteins and therefore have to be viewed with caution.

In a murine model of antigen-induced arthritis, immunization against and subsequent challenge with citrullinated vimentin induced stronger periarticular bone loss than immunization against and challenge with methylated bovine serum albumin (mBSA) (54). This effect was independent from inflammation, as mBSA induced more severe synovitis. Similarly, mice immunized with autologous citrullinated type II mouse collagen developed arthritis and bone loss correlating with serum ACPA levels (55).

Together, these studies indicate a direct effect of ACPA on osteoclastogenesis and bone loss. Whether this pro-osteoclastic effect is indeed based on antigen-antibody binding or is preferentially mediated by FcγR remains to be determined.

### Impact of Immune Complexes on Osteoclastogenesis

Recently, we found that under certain circumstances not only ACPA, but basically any kind of IgG containing immune complex can increase osteoclast number and bone resorption *in vitro* as well as *in vivo* via binding to FcγR (46, 56). In a murine model of inflammatory arthritis, the osteoclast specific deletion of FcγRIV resulted in a protection from aberrant osteoclast generation and bone erosion in inflamed joints, while inflammation itself was not affected, indicating that inflammatory cytokines alone are not sufficient to induce bone loss in inflammatory arthritis (47). Mice with a global deletion of the inhibitory FcγRIIB exhibit an osteoporotic phenotype even under steady state conditions due to an increase in osteoclast number (57). FcγRIIB is an important regulator of B cells and its deletion leads to a massive induction of autoantibodies (58) that could enhance osteoclastogenesis. Indeed, despite no difference in osteoclast numbers generated *ex vivo* from wildtype and FcγRIIB deficient bone marrow, the addition of sera from FcγRIIB-deficient mice resulted in an increased osteoclastogenesis. This effect could be blocked by IgG depletion or deletion of FcγRIII.

### Indirect Effects of Autoantibodies on Osteoclastogenesis by Induction of Proinflammatory Cytokines

**Figure 2:**
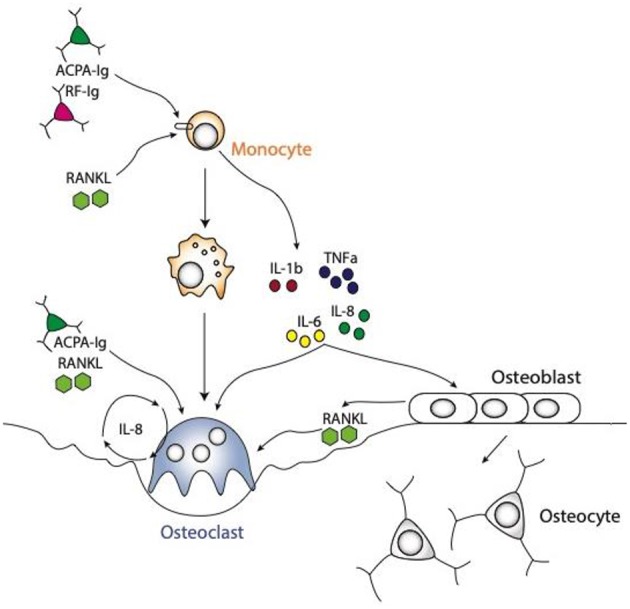
Schematic representations of osteoclast differentiation regulated by ACPA or RF antibodies induced cytokines produced by the monocytic lineages. The cytokines, like TNFa, IL1b, IL6, and IL8 are produced by monocyte lineage challenge with ACPA or RF autoantibodies. All these cytokines are directly able to enhance osteoclast formation. Moreover, they can also regulate stromal cells to secrete RANKL, which in turn will induce osteoclast differentiation. It is also well-known that ACPA can influence IL8 secretion by osteoclasts them-selves which will enhance osteoclastogenesis in an autocrine manner.

In addition to the direct action of autoantibodies on osteoclastogenesis, the release of inflammatory cytokines by macrophages upon autoantibody stimulation has been identified to enhance osteoclast differentiation and function (Figure 2). The disequilibrium between pro- and anti-inflammatory cytokine activities facilitates the induction of chronic inflammation and joint damage. It is less known though, how cytokines are organized within a hierarchical regulatory network. Macrophages are considered to play a seminal part in cytokine production in the joints of patients with RA and represent a major source for most of the prominent mediators of disease, such as tumor necrosis factor (TNF)-α and interleukin (IL)-6, but also other cytokines and chemokines involved in the disease process, such as IL-1β, IL-8, and chemokine (C-C motif) ligand 2 (CCL2) (59).

Autoantibodies and their immune complexes may play a central role in shaping a pro-inflammatory environment. Indeed, complexes of ACPA and RF induce robust cytokine production from human macrophages (60–62). This effect is mediated by FcγR signaling on macrophages inducing a strong activation signal for cytokine release (63). In particular, macrophages pre-exposed to M-CSF are sensitive to immune complex-mediated cytokine production. In the synovial membrane of RA patients, M-CSF is present in large amounts (64). We recently showed that treatment of human monocytes with ACPA antibodies or RF leads to the production of the cytokines TNF-α, IL-1β, IL-6, and IL-8 (65). This induction of pro-inflammatory cytokines can enhance osteoclast differentiation (Figure 2).

TNF-α is among the most potent cytokines to stimulate osteoclastogenesis. On one hand, TNF-α can induce TRAP positive cells in the absence of RANKL through the induction of the NF-κB pathway (66). On the other hand, TNF-α induces RANK expression by osteoclast precursors (67). In addition, TNF-α and RANKL cooperate to induce osteoclast formation in a TRAF-6 independent pathway through TRAF-3 signaling (68). In addition, TNF-α can indirectly regulate osteoclasts through various stimuli of the stromal cells, for examples by production of RANKL or other cytokines (69).

Like TNF-α, IL-6 is a powerful molecule to induce osteoclast differentiation (70). IL-6 binds the IL-6 receptor, comprising the subunit gp130 that is also required for other cytokines. IL-6R induction leads to STAT3 phosphorylation, followed by JAK, which finally induces osteoclast markers (70). Its role is quite contradictory, because one report described that IL-6 inhibits RANKL-induced osteoclastogenesis. It is likely that IL-6 independently regulates different pathways such as NF-κB, ERK or JNK, leading to alternative regulation of osteoclastogenesis (71, 72). In the treatment of human RA, TNF-α, and IL-6 antagonists ameliorate RA equally, indicating that both cytokines are key drivers of synovitis. Of note, the T cell costimulation inhibitor abatacept (cytotoxic T-lymphocyte associated antigen 4 (CTLA4) is most effective in patients with high ACPA and RF autoantibodies (73, 74). Tanaka et al. showed that immune complexes increased CD80/86 expression on monocyte lineages, rendering them sensitive to abatacept (75) which might explain the strong efficacy of abatacept in ACPA positive RA patients. Interestingly, abatacept treatment not only regulates monocytes but also osteoclast differentiation (76).

### Implications of Antibody Glycosylation

**Figure 3:**
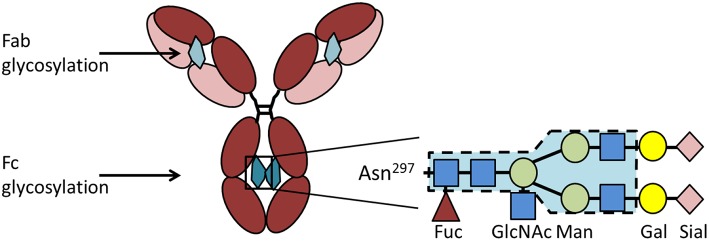
IgG glycosylation. IgG contains a conserved glycosylation site at the asparagine (Asn)^297^ in the CH2 domain of the heavy chain. The Fc glycan (depicted in petrol) consists of a conserved heptamer (shaded in blue) that can be extended by various additional sugar residues. Here we show a fully processed glycan containing all sugar residues possible. In addition to the Fc glycan, some IgG molecules contain a glycosylation site in the Fab region (depicted in light petrol) that occurs stochastically due to the introduction of a new glycosylation site during somatic hypermutation. Asn, asparagine; Gal, galactose; GlcNAc, N-acetylglucosamine; Fuc, fucose; Man, mannose; Sial, sialic acid.

IgG has one conserved Fc-glycosylation site located at asparagine-297 in the CH2 domain of the heavy chain (Figure 3). This glycosylation is critical for the correct conformation of the Fc part and regulates the binding affinity of IgG to FcγR [reviewed in Arnold et al. (77)]. Elimination of the glycan either by enzymatic deglycosylation or by mutation of asparagine-297 to alanine results in a loss of FcγR binding and hence effector functions (78–80). The glycan core structure is strongly conserved and consists of a heptamer of mannose and N-acetyl glucosamine residues. This core structure can be extended by galactose, terminal sialic acid, bisecting N-acetylglucosamine, and core fucose, resulting in a huge variety of theoretically possible glycoforms [reviewed in Zauner et al. (81)]. The exact composition of the Fc glycan determines whether IgG exerts rather pro- or anti-inflammatory effects on immune cells. Especially galactose and terminal sialic acid have been shown to render IgG more anti-inflammatory. 35–45% of random serum IgG from healthy donors is monogalactosylated and 16–27% is bigalactosylated (82). Galactosylation decreases with age (83). During pregnancy, galactosylation is increased and correlates with pregnancy-induced remission of RA (84–86). Only about 10–20% of human serum IgG is sialylated (87, 88), but this low percentage seems to be enough to sustain an anti-inflammatory environment under healthy conditions. It is believed that sialylated IgG actively suppresses immune cells via receptors of the C-type lectin superfamily, such as dendritic cell specific ICAM-grabbing non-integrin (DC-SIGN) (with the murine ortholog SIGNR-1) and the dendritic cell immunoreceptor (DCIR) (89, 90). In addition to Fc glycosylation, about 15–25% of IgG contain Fab glycosylation sites [reviewed in Zauner et al. (81)]. These sites emerge during somatic hypermutation. So far it is unclear if Fab glycosylation has a functional role.

Of note, ACPA display less terminal sialic acid compared to total IgG. ACPA from synovial fluid are even less sialylated (91). The low sialic acid content of ACPA and probably also of other autoantibodies seems to play a key role for the development of clinical disease and bone erosion. In a murine model of collagen induced arthritis, we found that mice fed with sialic acid precursor N-acetylmannosamine did not only display higher sialylation of IgG1, but also have a lower incidence, lower arthritis scores and less bone destruction (46). In addition, it was shown that mice lacking IL-23 do not develop collagen-induced arthritis despite the induction of collagen autoantibodies (92). Autoantibody titers and affinity were not changed compared to wildtype mice, but autoantibodies from IL-23 deficient mice contained more sialic acid. Enzymatic removal of terminal sialic acid resulted in higher arthritis scores, demonstrating the importance of antibody glycosylation for IgG activity. The importance of glycosylation for autoantibody-mediated bone loss is further demonstrated by the fact that even pooled serum IgG from healthy donors is able to enhance osteoclastogenesis and bone resorption after complexation and enzymatic removal of sialic acid (46).

So far it is not completely understood how antibody glycosylation is regulated. The IL-23-T_H_17 axis seems to play a crucial role in autoantibody sialylation by the regulation of the enzyme ST6 beta-galactoside α-2,6-sialyltransferase 1 (St6Gal1) that attaches sialic acid to terminal galactose residues (92). Also estrogen positively regulates St6Gal1 expression and postmenopausal women with RA receiving hormone replacement therapy displayed significantly increased Fc sialylation of IgG (93).

### Conclusions and Future Research Agenda

Within the last years, evidence emerged that disease-associated autoantibodies play an important role in the development of bone loss in RA. Especially ACPA have been shown to contribute to aberrant osteoclast formation and activation either by direct stimulation of osteoclast precursors or the induction of a cytokine storm mainly by macrophages. Furthermore, low Fc sialylation of ACPA contributes to their inflammatory and pro-osteoclastogenic phenotype.

The majority of studies addressing autoantibody-mediated bone loss has been performed in mice or *in vitro* models and might incompletely reflect the human situation. There are interesting human studies suggesting that ACPA induce bone loss in the absence of inflammation. However, additional human studies are needed to clarify to what extent ACPA contribute to bone loss in RA patients.

Furthermore, beside the hyperactivation of osteoclasts, an impairment of osteoblast development and function is found in RA patients that aggravates bone loss. To date there is no report showing a direct action of ACPA or RF autoantibodies on osteoblast differentiation or activity, although the FcγRs have been shown to be expressed by stromal cells (47). It would be essential to further develop *in vivo* and *in vitro* experiments delineating the molecular actions of autoantibodies on bone formation.

Beside ACPA, a variety of antibodies directed against modified proteins (AMPA) have been discovered in the last years. Most prominent are anti-CarP, but also antibodies against proteins that have undergone acetylation, oxidation, or malondialdehyde-acetaldehyde addition have been described in RA patients [reviewed in Chang and Nigrovic (94)]. It is likely that other autoantibodies against posttranslational modifications, such as anti-CarP affect osteoclastogenesis and bone loss as well. However, there are no mechanistic data available so far. In addition, there are other autoantibodies known that act via completely different mechanisms than the ones described in this article. For example autoantibodies against OPG function as enhancers of osteoclastogenesis by neutralizing OPG [reviewed in Hauser and Harre (95)]. These autoantibodies are found in autoimmune diseases, such as RA and celiac disease and seem to be a feature that is independent of the original disease drivers. Nevertheless, it might be important to check for the presence of these autoantibodies, especially in patients that are not responding to the current therapies.

Based on the fact that ACPA are associated with increased bone loss in RA, one would wish to control autoimmunity in RA and to induce seroconversion or at least lowering of ACPA levels by treatment. Such approaches are for instance B cell depletion by rituximab or inhibition of T cell co-stimulation with abatacept, which are approved therapies in RA and which significantly lower ACPA levels (96). However, whether modifying autoantibody levels has clinical value in controlling disease, remains to be determined. Aside from antibody reduction, the triggers promoting the induction of effector function of autoantibodies are also targets for future interventions. Given the fact that Fc glycosylation (and especially sialylation) controls the pathogenicity of autoantibodies, it will be interesting to know whether one can control IgG sialylation in RA patients. This might be an important step toward tolerance induction and disease cure.

In addition, in the last years, more attention has been laid on the role of the mucosal immune system during RA initiation and propagation [reviewed in Caminer et al. (97) and Wells et al. (98)]. Dysregulations of microbiota and the gut barrier function might trigger the series of events that deregulate T and B cell responses resulting in autoimmunity in RA patients.

## Author Contributions

All authors listed have made a substantial, direct and intellectual contribution to the work, and approved it for publication.

### Conflict of Interest Statement

The authors declare that the research was conducted in the absence of any commercial or financial relationships that could be construed as a potential conflict of interest.
